# Therapeutic Effects of Intravenous Injection of Fresh and Frozen Thawed HO-1-Overexpressed Ad-MSCs in Dogs with Acute Spinal Cord Injury

**DOI:** 10.1155/2019/8537541

**Published:** 2019-08-01

**Authors:** Imdad Ullah Khan, Yongseok Yoon, Kyeung Uk Choi, Kwang Rae Jo, Namyul Kim, Eunbee Lee, Wan Hee Kim, Oh-Kyeong Kweon

**Affiliations:** BK21 PLUS Program for Creative Veterinary Science Research, Research Institute for Veterinary Science and College of Veterinary Medicine, Seoul National University, 151-742 Seoul, Republic of Korea

## Abstract

Owing to the antioxidant and anti-inflammatory functions of hemeoxygenase-1 (HO-1), HO-1-expressing canine adipose-derived mesenchymal stem cells (Ad-MSCs) could be efficacious in treating spinal cord injury (SCI). Further, frozen thawed HO-1 Ad-MSCs could be instantly available as an emergency treatment for SCI. We compared the effects of intravenous treatment with freshly cultured HO-1 Ad-MSCs (HO-1 MSCs), only green fluorescent protein-expressing Ad-MSCs (GFP MSCs), and frozen thawed HO-1 Ad-MSCs (FT-HO-1 MSCs) in dogs with acute SCI. For four weeks, dogs were evaluated for improvement in hind limb locomotion using a canine Basso Beattie Bresnahan (cBBB) score. Upon completion of the study, injured spinal cord segments were harvested and used for western blot and histopathological analyses. All cell types had migrated to the injured spinal cord segment. The group that received HO-1 MSCs showed significant improvement in the cBBB score within four weeks. This group also showed significantly higher expression of NF-M and reduced astrogliosis. There was reduced expression of proinflammatory cytokines (IL6, TNF-*α*, and IL-1*β*) and increased expression of anti-inflammatory markers (IL-10, HO-1) in the HO-1 MSC group. Histopathological assessment revealed decreased fibrosis at the epicenter of the lesion and increased myelination in the HO-1 MSC group. Together, these data suggest that HO-1 MSCs could improve hind limb function by increasing the anti-inflammatory reaction, leading to neural sparing. Further, we found similar results between GFP MSCs and FT-HO-1 MSCs, which suggest that FT-HO-1 MSCs could be used as an emergency treatment for SCI.

## 1. Introduction

Presently, a high dose of methylprednisolone sodium succinate (MPSS) is injected intravenously (IV) as an emergency treatment to induce antioxidation in dogs with acute spinal cord injury (SCI). However, a meta-analysis has demonstrated that there is no satisfactory improvement with the use of MPSS, and it is associated with adverse side effects such as severe gastrointestinal bleeding [1, 2]. Thus, a safer and more effective emergency treatment option is required. An alternative that has been attempted is IV administration of adipose-derived mesenchymal stromal cells (Ad-MSCs). Ad-MSCs and MPSS have different roles in controlling oxidative stress and inflammation in dogs with acute SCI [3]. Owing to the strong antioxidant and anti-inflammatory effects of hemeoxygenase-1 (HO-1), intraparenchymal injection of HO-1-expressing Ad-MSCs reduces inflammation and improves neuronal sparing in canine SCI compared to control Ad-MSCs.

HO-1 is an antistress enzyme which causes oxidative degradation of deleterious heme into biologically active products such as carbon monoxide, biliverdin, and ferrous iron [4]. Excessive intracellular iron accumulation induces the production of reactive oxygen species (ROS) which causes apoptosis in bone marrow MSCs. HO-1 decreases apoptosis by halting the production of ROS through upregulating IL-10 [5]. HO-1 causes immunomodulation by altering the function of macrophages, regulatory T cells, and dendritic cells [6]. HO-1 is normally expressed in neurons in the spinal cord [7]. During SCI, the expression of HO-1 is induced in glial cells, which prevents inflammatory damage to the neurons [8]. Due to the concerted actions of HO-1, the therapeutic effects of HO-1 have been evaluated in various inflammatory conditions, neuropathological disorders, and cardiovascular diseases [9]. Adeno-associated HO-1 induction following SCI causes neuroprotection by inhibiting proapoptotic pathways in the neurons [10]. It protects against neuronal damage due to ischemia-reperfusion injury by inducing the expression of antioxidant enzymes such as Cu and Zn superoxide dismutase and neurotrophic factors such as BDNF and inhibiting lactic acidosis [11]. In SCI and cardiovascular disease, transplantation of mesenchymal stem cells (MSCs) modified by viral-mediated HO-1 gene insertion shows improved healing due to the stimulated expression of anti-inflammatory cytokines and vascular endothelial growth factor [12, 13]. HO-1 induction is necessary to limit the pathogenesis of diabetes mellitus by reducing excessive ROS, low-density lipoproteins, and proinflammatory cytokines [14].

MSCs have been administered through various routes such as IV [3, 15], intraparenchymal [13, 16], and into the subarachnoid [17] and epidural spaces [18]. HO-1-overexpressed bone marrow MSCs have avoided acute allogeneic small intestine graft rejection after IV injection through suppressing NK cell activity, inflammation, and apoptosis [19]. The IV route is simple and minimally invasive, with favorable clinical outcomes, and avoids complications associated with the other relatively invasive procedures used for cell transplantation [20].

Cryopreservation of cells could lead to more instantaneous availability for use after SCI, and frozen thawed HO-1-overexpressed stem cells could be the best option for critical care. In the present study, we evaluated whether IV injection of freshly cultured and frozen thawed HO-1-overexpressed Ad-MSCs is effective as an emergency treatment in dogs with acute SCI.

## 2. Materials and Methods

### 2.1. *In Vitro* Procedures

#### 2.1.1. Isolation and Culture of Ad-MSCs

Adipose tissue was collected aseptically from the subgluteal region of a healthy beagle dog under general anesthesia. The tissue was homogenized and incubated with collagenase type 1 enzyme (1 mg/mL, Sigma, St. Louis, MO, U.S.A.) for 2 h at room temperature (16-20°C). The suspension was filtered through a 100 *μ*m nylon mesh then centrifuged at 4°C for 10 min. The supernatant was discarded, and the stromal vascular fraction (SVF) was carefully isolated. The SVF was resuspended with phosphate-buffered saline (PBS) and cultured in a 150 mm Petri dish containing low-glucose Dulbecco's modified Eagle's medium (DMEM; GenDEPOT, Grand Island, NY, U.S.A.) with 10% fetal bovine serum (FBS; Gibco BRL, Grand Island, NY, U.S.A.) and 1% penicillin and streptomycin (PS). Cells were washed with PBS after 24 h of incubation to remove the dead cells and tissue debris, and media were changed. The cells were subcultured when 90% confluence was reached. The third passage (P3) of Ad-MSCs was used in subsequent experiments [21]. These cells have been previously confirmed for their multiline differentiation capability [16, 22].

#### 2.1.2. Lentivirus-Mediated HO-1 Gene Insertion into Ad-MSCs

A Dharmacon trans-lentivirus packaging system (GE Healthcare, Lafayette, CO, U.S.A.) was used to produce HO-1-overexpressing Ad-MSCs. Two types of cells were produced: GFP-expressing Ad-MSCs (GFP MSCs) and GFP-HO-1-expressing Ad-MSCs (HO-1 MSCs).

According to the manufacturer's guidelines, we first amplified the flag-tagged HO-1 gene by using Phusion DNA Polymerases (Thermo Scientific, Pittsburgh, PA, U.S.A.) from the cDNA of canine peripheral blood. The HO-1 primer (forward GACAGCATGCCCCAGGAT, reverse CACAGCCTAAGGAGCCAGT) specific for canines was inserted into the pCDH-EF1-MCS-pA-PGK-copGFP-T2A-Puro vector using the restriction enzymes EcoRI and BamHI (System Biosciences, Mountain View, CA, U.S.A.) [23]. HEK293T cells were cultured in a 100 mm culture dish. Culture medium DMEM containing high glucose, 10% FBS, and 1% PS was used to grow the cells at 37°C and 5% CO_2_. When the cells reached 90% confluence, the vector encoding GFP and HO-1 genes was mixed with lentiviral packaging mix (Fisher Scientific Cat#14-432-23) and added dropwise to the cultured HEK293T cells. The cells were cultured for 48 h at 37°C and 5% CO_2_. The medium was changed once after 16 h of incubation, and viral particles were collected from the culture media after 48 h of incubation. Hence, two types of viruses were produced: Lenti-GFP and Lenti HO1-GFP. Using the manufacturer guidelines, the concentration of viral particles was determined. The second passage Ad-MSCs were infected with lentiviruses at MOI 100 [24] at 50-60% confluence. At 90% confluence, the cells were subcultured. Successful transduction was confirmed by the expression of GFP under a fluorescent microscope (EVOS FL imaging system, Stanwood, Washington, U.S.A.), quantitative polymerase chain reaction (qPCR), and western blot analysis. The cells were treated with 3 *μ*g/mL puromycin to obtain a higher percentage of transduced stem cells. Finally, we got two types of cells: Ad-MSCs expressing GFP (GFP MSCs) and Ad-MSCs expressing GFP and HO-1 (HO-1 MSCs). The transduced Ad-MSCs were cultured to P3 and used in the subsequent experiments.

#### 2.1.3. Real-Time Quantitative PCR (rtPCR)

The transduced Ad-MSCs were analyzed for the expression of transfected genes by qPCR and western blot analysis. The cells were cultured in four 150 mm culture dishes until they reached 90% confluence. One culture dish was utilized for qPCR, and 3 culture dishes were utilized for western blot analysis. For qPCR, mRNA was harvested using Hybrid-R RNA extraction kit (GeneAll, Seoul, Republic of Korea) according to manufacturer guidelines. Total mRNA was determined by Gen 5.2 reader type, Epoch (BioTek, Winooski, Vermont, VT, U.S.A.), by measuring optical density at a wavelength of 260 nm. cDNA was synthesized by using Prime Script II First-Strand cDNA Synthesis Kit (Takara, Otsu, Japan). cDNA was amplified by ABI StepOnePlus Real-Time PCR System (Applied Biosystems, Foster City, California, U.S.A.) after mixing it with SYBR premix EX Taq (Takara, Otsu, Japan) and the primers (forward, reverse) for NANOG, OCT-4, SOX2, IL-6, TNF-*α*, IL-10, HO-1, and BDNF (Table 1). The glyceraldehyde 3-phosphate dehydrogenase (GAPDH) gene was used as a reference gene (Table 1). The relative gene expression was quantified by the 2^-*ΔΔ*CT^ method [25].

The western blot analysis was performed to detect the expression of HO-1 by HO-1 MSCs and FT-HO-1 MSCs relative to GFP MSCs. For this purpose, cells from three culture dishes were scraped and centrifuged. In the case of FT-HO-1 MSCs, the cells were obtained from 3 cryovials and centrifuged. The cell pellet was resuspended and incubated with 300 *μ*L RIPA Lysis Buffer (GenDEPOT, Grand Island, NY, U.S.A.) for 30 min on ice. The suspension was centrifuged at 4°C for 10 min with 13,000 rpm. The supernatant containing the proteins was separated and mixed with proteinase inhibitor solution (GenDEPOT, Grand Island, NY, U.S.A.) at the rate of 1 *μ*L/100 *μ*L of cell lysate. Protein concentration was measured by the Bradford assay [26]. Approximately 20 *μ*g of protein was separated by 10% sodium dodecyl sulfate-polyacrylamide gel electrophoresis (SDS-PAGE) and then transferred to a polyvinylidene difluoride (PVDF) membrane. The membrane was blocked and incubated with primary antibodies for GFP (GF28R), *β*-actin (sc-47778), and HO-1 (ab-13243), followed by incubation with secondary antibodies anti-mouse secondary antibody (ab6728) and anti-rabbit secondary antibody (ab6721). The protein bands were visualized with enhanced chemiluminescent substrates (ECL) (Bio-Rad, Hercules, California, U.S.A.), by using image quant LAS 4000 mini system (GE Healthcare Biosciences, Uppsala, Sweden).

#### 2.1.4. Measurement of Antioxidant Concentration

The antioxidant concentration was measured by Antioxidant Assay Kit (Sigma-Aldrich, St. Louis, MO, U.S.A.). Following the manufacturer's guidelines, the cell lysate was prepared from HO-1 MSCs, GFP MSCs, and FT-HO-1 MSCs. The cell lysate was incubated with myoglobin and ABTS substrate working solution for 5 min. The reaction was stopped with a stop solution, and the optical density was measured at 405 nm. The antioxidant concentration was measured through a Trolox standard curve in millimole (mM) units.

#### 2.1.5. Cryopreservation of HO-1 MSCs

P3 HO-1 MSCs cultured in a 150 mm culture plate were harvested at 90% confluence. The cells were washed twice with PBS and incubated with 0.05% trypsin-EDTA for 15 min at 37°C and 5% CO_2_ (Sigma-Aldrich, St. Louis, MO, U.S.A.). The cells were then centrifuged at 2,500 rpm for 5 min at 4°C and the supernatant was discarded. Approximately 5 × 10^6^ cells were resuspended in the cryogenic medium (50% DMEM, 40% FBS, and 10% dimethyl sulfoxide) and transferred into a cryovial (1.2 mL, Sigma-Aldrich, St. Louis, MO, U.S.A.). The cells were kept at 4°C for 1 h, -20°C for 2 h, -80 C for 24 h, and -150°C for 2-3 weeks [23].

### 2.2. *In Vivo* Procedures

#### 2.2.1. Selection and Identification of Animals

The study was performed on 12 healthy beagle dogs aged 1.2 ± 0.2 years and weighing 12 ± 3.0 kg. This study was approved by the Institute of Animal Care and Use Committee (ICAUC) of Seoul National University (SNU-170417-12). The dogs were randomly divided into four groups comprising four dogs each. The groups were named according to the type of cells transplanted IV: GFP MSCs (fresh Ad-MSCs expressing GFP), HO-1 MSCs (fresh Ad-MSCs expressing GFP and HO-1), and FT-HO-1 MSCs (frozen thawed HO-1 Ad-MSCs).

#### 2.2.2. Induction of Spinal Cord Injury

SCI was induced by hemilaminectomy as previously described [27]. Dogs were restrained in the ventral recumbent position under general anesthesia. A small opening was made using a pneumatic burr between the third and fourth lumbar vertebrae. Under fluoroscopic guidance, a 6-silicone Foley catheter (Yushin, South Korea) was inserted into the spinal canal and dragged up to the 1^st^ lumbar vertebra (L1), until its tip reached the cranial border of L1. The catheter balloon was inflated with contrast media (Omnipaque, Amersham Health, Carrington Hill, Ireland) at a rate of 50 *μ*L/kg. The catheter was deflated and removed after 6 h. The dogs were closely evaluated for the presence of sensory and motor reflexes. Only the dogs which showed complete paraplegia, with no sensory and motor reflexes, were included in the study. All dogs were kept in an intensive care unit for 3 days. Urination was assisted by manual bladder compression three times per day. Soft bedding was provided to prevent bed sores, and food and water were provided ad libitum.

#### 2.2.3. Transplantation of Ad-MSCs

Stem cells were transplanted immediately after the removal of the embolectomy catheter. In the case of transplantation of fresh MSCs, the cells were cultured in a 150 mm culture dish until they had reached 90% confluence. The cells were harvested, centrifuged, and pelleted as described above. In the case of transplantation of frozen thawed MSCs, the cryopreserved cells were thawed by warming the cryovial at 37°C for 5 min. The cell suspension was mixed with an equal volume of DMEM and centrifuged at 2,500 rpm for 5 min at 4°C. The medium was discarded and the cell pellet was resuspended in PBS and centrifuged. The cell pellets obtained from both fresh and frozen thawed preparations were resuspended in 1 mL of Hartmann's solution. The cell number required for transplantation was counted using a Countess FL Automated Cell Counter (Thermo Fisher Scientific, Pittsburg, PA, U.S.A.) after staining with Trypan blue. The %age of GFP expression for HO-1 MSCs, GFP MSCs, and FT-HO-1 MSCs was 91.03 ± 0.8, 91.30 ± 0.7, and 90.56 ± 0.95, respectively. About 1 × 10^7^ fresh GFP MSCs and HO-1 MSCs and 1.5 × 10^7^ frozen thawed HO-1 MSCs were diluted in 20 mL of Hartmann's solution and injected by slow IV infusion in 10 min. The cells were injected IV for three consecutive days with an interval of 24 h between each injection. The dogs were kept for four weeks after cell transplantation.

#### 2.2.4. Behavioral Assessment

All the dogs were observed for the improvement in hind limb movements every week for four weeks. The assessment was made by three people blinded to the study, and the score for each week was presented as an average. The motor activity of hind limbs was assessed by using the canine Basso Beattie Bresnahan (cBBB) scale [28]. Each week, the dogs were released into a confined area for 5 min and observed from each side to note their ability to use the hind limbs. Hind limb activity was observed when the dogs were at rest, walking, and forced walking. The hind limb joints were flexed while the dogs were sitting and when lifted up, to observe the reflex motor response and movements of the joints. Deep pain perception was evaluated by pinching the phalangeal joints with artery forceps [29].

#### 2.2.5. Harvesting of the Spinal Cord and Sample Preparation

At the end of the four-week experimental period, the dogs were anesthetized and the spinal cords were harvested from T13 to L2. The spinal cord segments containing the entire injured segment were put into 10% sucrose for 24 h and then into 20% sucrose for the subsequent 24 h. The dura mater was carefully removed, and the injured spinal cord segments were frozen in OCT compound using liquid nitrogen. The injured spinal cord segments were carefully sliced from the center into two equal longitudinal halves. One half was used for western blotting while the other half was used for histopathology.

#### 2.2.6. Western Blot Analysis for the Expression of Proteins

The injured spinal cord segments were crushed and minced by hammering after freezing with liquid nitrogen. Crushed tissue was then incubated with RIPA lysis buffer and proteinase inhibitor solution for 30 min according to the manufacturer's guidelines. Samples were centrifuged at 13,000 rpm for 15 min at 4°C. The supernatant was collected into a separate Eppendorf tube, and the protein concentration was determined by a Bradford assay. Approximately 20 *μ*g of protein was separated through 10% acrylamide gel by a water tank gel electrophoresis. The proteins were electrically transferred onto a PVDF membrane. The membrane was blocked with 20% skim milk for 1 h and washed. Membranes were then incubated with primary antibodies for 16-24 h. The primary antibodies specific for neural markers Tuj-1 (sc69966, *β*-III tubulin), NF-M (sc-398532, neurofilament), and nestin (ab7695, neural progenitor stem cells) and glial markers GFAP (sc65343, astrocytes), GALC (sc67352, galactosylceramidase), and CD11b (ab62817 microglia) were used. To stain for inflammatory cytokines, the following primary antibodies were used: TNF-*α* (sc1350, tumor necrosis factor alpha), IL6 (ab6672, interleukin-6), and IL1-*β* (A13268, interleukin 1*β*). To stain for anti-inflammatory markers, the following primary antibodies were used: IL-10 (R&D, MAB7352) and HO-1 (ab13243). The primary antibody for GFP (GF28R) was used to assess the migration of transplanted cells into the injured segment of the spinal cord. The membrane was then incubated for 2 h at 4°C with the anti-mouse secondary antibody (ab6728), anti-rabbit secondary antibody (ab6721), and anti-goat secondary antibody (sc2354). *β*-Actin was used as a reference antibody (sc47778). Enhanced chemiluminescent substrates (ECL) (Bio-Rad, Hercules, California, U.S.A.) were used to visualize the protein bands, and images were quantified using the LAS 4000 mini system (GE Healthcare, Lafayette, Co, U.S.A.).

#### 2.2.7. Immunocytochemistry

The remaining half of the spinal cord was cryosectioned at -20°C. The segments containing the injury were sliced at 10 *μ*m and mounted on silane-coated slides. OCT compound was carefully removed by pouring phosphate-buffered saline (PBS) on the slides, followed by fixing with 4% paraformaldehyde for 10 min, then permeating with 0.1% *v*/*v* Triton X-100 for 3 min. Slides were blocked with 10% FBS (FBS, Gibco BRL, Grand Island, NY, U.S.A.) in PBS for 1 h, washed, and incubated overnight with primary antibodies for GFP, Tuj-1, NF-M, nestin, GFAP, and GALC at 4°C. Slides were washed and incubated with fluorescein iso-thio-cyanate conjugated anti-rabbit (Flamma 648) and anti-mouse (Alexa flour, ab-150111) secondary antibodies. One or two drops of DAPI were added to the sections to stain the nuclei, and slides were coverslipped. The sections were examined for the expression of relevant markers with a fluorescent microscope (EVOS FL imaging system, Stanwood, Washington, U.S.A.). The injured segments of the spinal cord were examined at low magnification 100x, and the cells stained positive for the specific marker were counted in the area of 0.75cm^2^ centered at the injured spinal cord segment, through an integrated cell counting system in the microscope software. The values were presented as a percentage and as an average for the relative group.

#### 2.2.8. Histopathological Assessment

Histopathology was performed to assess the degree of fibrosis, demyelination, hemorrhage, and vacuole formation. Following the manufacturer guidelines, slides were stained with hematoxylin and eosin (Thermo Fisher Scientific, Waltham, MA, U.S.A.) to assess the degree of fibrosis and with luxol fast blue (American MasterTech, Lodi, CA, U.S.A.) to assess the degree of demyelination. Slides were observed at 40x (scale bar 1,000 *μ*m) [30]. About 2-3 images that comprised the entire injured segment were obtained, and the lesion area was measured using ImageJ software (Version 1.37; National Institutes of Health, Bethesda, MD, U.S.A.). Values are presented as the average for each slide of a corresponding group.

#### 2.2.9. Statistical Analysis

Data are expressed as mean ± standard error (SE) and analyzed for the presence of significance by nonparametric Kruskal Wallis test. A Man-Whitney *U* test was used to determine significance between two specific groups. SPSS software (version 23 IBM, Chicago, IL, U.S.A.) was used to analyze the data, and a *p* value ≤0.05 was considered significant.

## 3. Results

### 3.1. HO-1 Gene Transfection and the Biological Characteristics of HO-1 MSCs

**Figure 1:**
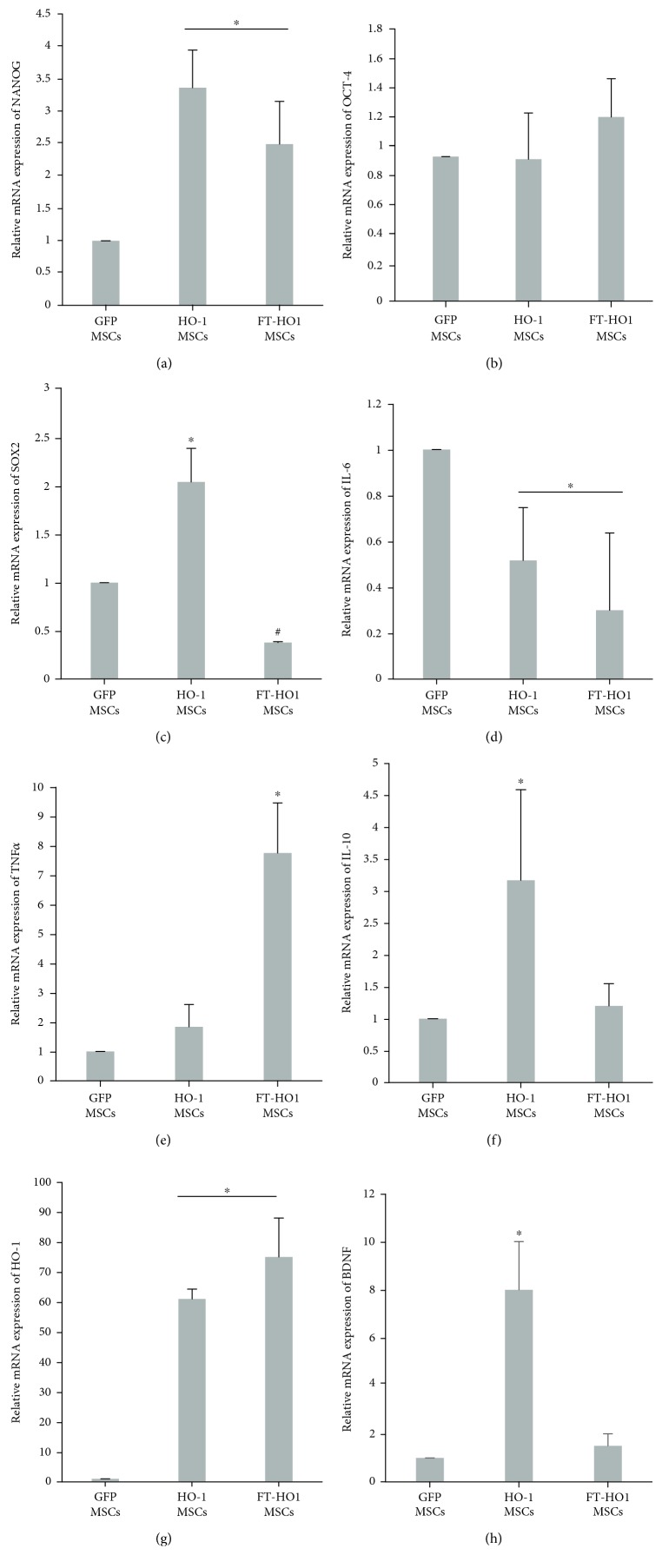
mRNA expression of factors (a) NANOG, (b) OCT-4, (c) SOX2, (d) IL-6, (e) TNF-*α*, (f) IL-10, (g) HO-1, and (h) BDNF. Each bar represents the average of four samples and error bars represent standard error. ∗ and # indicate significance at *p* ≤ 0.05.

**Figure 2:**
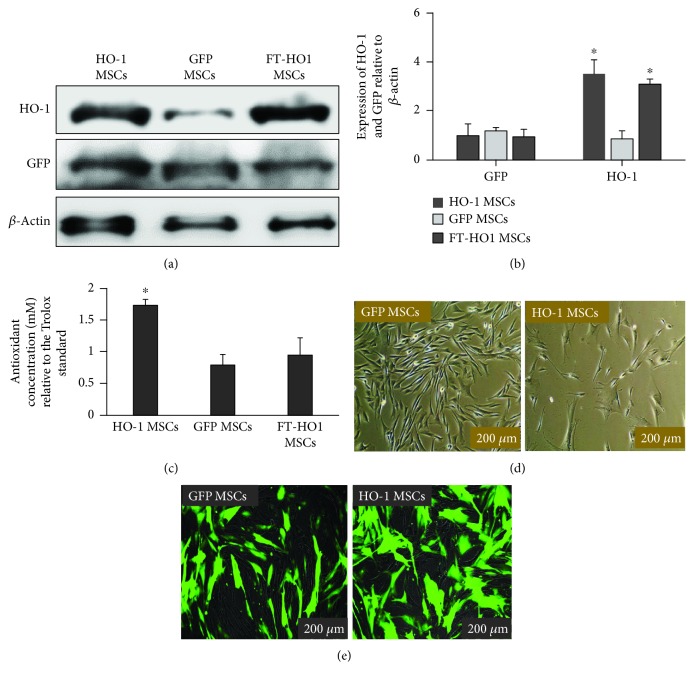
HO-1 and GFP gene transfection in Ad-MSCs. (a) Representation of the densities of HO-1 and GFP proteins obtained from a cell lysate of HO-1 and GFP MSCs. (b) Quantitative expression of densities obtained by western blot relative to *β*-actin. Each bar represents the average of six samples and error bars represent standard error. (c) Measurement of antioxidant concentration (mM) compared to Trolox standard curve. Each bar represents the average of six samples and error bars represent standard error. ^∗^Indicates significance at *p* ≤ 0.05 (Student *t-*test). (d) Positive GFP expression by GFP and HO-1 MSCs.

HO-1 gene was successfully transfected into Ad-MSCs. The mRNA gene expression of HO-1 in HO-1 MSCs was significantly higher (Figure 1(g),^∗^*p* ≤ 0.05) than that of GFP MSCs. Similarly, at a translation level, the cell lysate obtained from HO-1 MSCs contained higher levels of HO-1 protein (Figures 2(a) and 2(b), ^∗^*p* ≤ 0.05) than that from GFP MSCs. The expression of GFP was not significantly different between HO-1 MSCs and GFP MSCs (Figures 2(b) and 2(e)). GFP MSCs showed thin elliptical fibroblast-like morphology, while the HO-1 MSCs have a slightly broader elliptical shape (Figures 2(d) and 2(e)). The GFP MSCs showed slightly high proliferation, which was lower in HO-1 MSCs (Figure 2(d)).

Owing to the strong antioxidant functions of HO-1, the HO-1 MSCs were evaluated for their antioxidant capability. The antioxidant concentration determining the total antioxidant capacity was significantly higher in HO-1 MSCs than in GFP MSCs and FT-HO-1 MSCs; however, it was the same between GFP MSCs and FT-HO-1 MSCs (Figure 2(c), ^∗^*p* ≤ 0.05).

The genes related to stemness such as NANOG, OCT-4, and SOX2 were not negatively regulated in HO-1 MSCs. NANOG was significantly upregulated in both HO-1 MSCs and FT-HO-1 MSCs (Figure 1(a), ^∗^*p* ≤ 0.05); however, the expression of SOX2 was downregulated in FT-HO-1 MSCs (Figure 1(c), ^#^*p* ≤ 0.05). The expression of OCT-4 remained the same among all the groups (Figure 1(b)). Regarding the expression of inflammatory gene markers, IL-6 was downregulated in both HO-1 MSCs and FT-HO-1 MSCs compared with GFP MSCs (Figure 1(d), ^∗^*p* ≤ 0.05). The expression of TNF-*α* was higher in FT-HO-1 MSCs, but it was same between HO-1 MSCs and GFP MSCs (Figure 1(e), ^∗^*p* ≤ 0.05). Comparing the expression of anti-inflammatory factors, IL-10 was only upregulated in the HO-1 MSCs (Figure 1(f), ^∗^*p* ≤ 0.05); however, HO-1 was highly upregulated in both HO-1 and FT-HO-1 MSCs (Figure 1(g), ^∗^*p* ≤ 0.05). The gene expression of the neurotropic factor such as BDNF was upregulated only in HO-1 MSCs compared to GFP MSCs and FT-HO-1 MSCs (Figure 1(h), ^∗^*p* ≤ 0.05).

### 3.2. Migration of Cells after IV Injection

**Figure 3:**
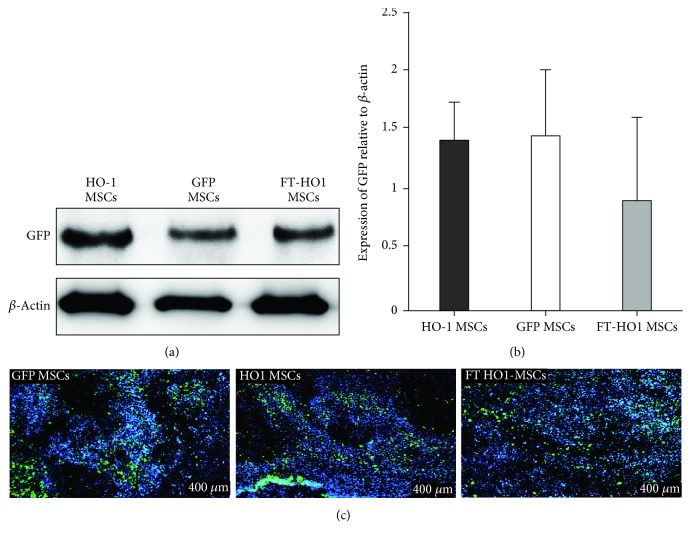
Migration of injected cells at the injured spinal cord segment following 4 weeks of experimental period. (a) Representative densities of GFP protein in groups obtained through western blot analysis of tissue lysate. (b) Depiction of the quantitative expression of GFP protein relative to *β*-actin. Each bar represents the average of 4 samples in a group and error bars represent standard error. (c) Immunostaining for the location of transplanted cells at the injured spinal cord segment. The nuclei are stained blue and GFP protein is stained green, demonstrating the migration of transplanted cells. Each figure is obtained with a scale bar of 400 *μ*m.

After IV cell transplantation, no immediate or delayed hypersensitivity reaction was observed. Based upon western blot analysis and immunocytochemistry, cells from all groups had successfully migrated into the injured spinal cord segment after IV injection (Figure 3). Regarding the expression of GFP in vivo, no significant difference was observed among the groups (Figures 3(a)–3(c)). As indicated by the positive expression of GFP detected using immunocytochemistry (Figure 3(c)), the GFP-positive cells were found in abundance at the rostral and caudal margins of the injured spinal cord segment; however, their expression was comparatively lower in the medial area of the injured spinal cord segment. More cells were present in gray matter at the rostral, medial, and caudal sites of the injured spinal cord segment compared to white matter (Figure 3(c)). The percentages of GFP expression quantified were 12.18% ± 2.84, 10.85% ± 3.03, and 8.34% ± 4.04 for HO-1 MSCs, GFP MSCs, and FT-HO-1 MSCs, respectively.

### 3.3. Functional Recovery

Recovery in hind limb locomotion was assessed using the 19-point cBBB scoring system. After SCI induction, dogs were closely evaluated for the presence of deep pain and joint movement. Before cell transplantation, the cBBB score of all dogs was zero. The hind quarter was completely paralyzed with no pain perception or movement in the joints. Defecation was normal in all the dogs, but they failed to urinate voluntarily.

**Figure 4:**
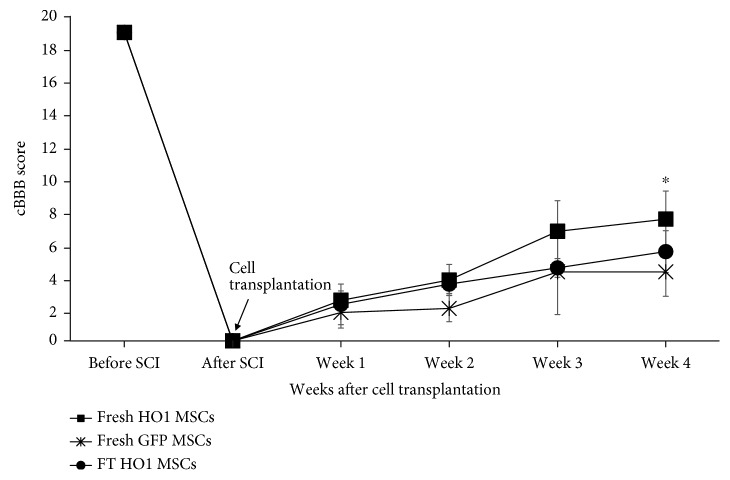
Improvement in cBBB score during the 4 weeks following cell transplantation. ^∗^Indicates significance at *p* ≤ 0.05 among the groups. Each point represents the average of 4 samples and error bars represent standard error.

After cell transplantation, the dogs showed gradual improvement in the motor recovery. The HO-1 MSC group showed rapid locomotor recovery in 4 weeks, demonstrated by a significantly higher cBBB score compared to the GFP MSC and FT-HO-1 MSC groups (Figure 4, ^∗^*p* ≤ 0.05). The improvement in cBBB score observed after 4 weeks was 7.5 ± 1.70, 4.25 ± 1.50, and 5.5 ± 1.29 for the HO-1 MSC, GFP MSC, and FT-HO-1 MSC groups, respectively. The HO-1 MSC group showed extensive movements of all the joints. Two of the dogs could support their body weight and attempted to stand when feeding or undergoing physical assessment. When they were forced to walk, one dog failed to support his body weight while the other dog could weakly lift his hind limb, with dorsal stepping and incoordination between hind limbs. No dog in either of the other groups could lift their body weight either at rest or during assisted walking. No dog showed recovery of pain perception. The dogs did not vocalize or react while their phalanges were pinched with artery forceps. Voluntary urination was recovered only in 2 dogs of the HO-1 MSC group, 1 dog of the GFP MSC group, and none in the FT-HO-1 MSC group.

### 3.4. Neural Sparing and Astrogliosis

**Figure 5:**
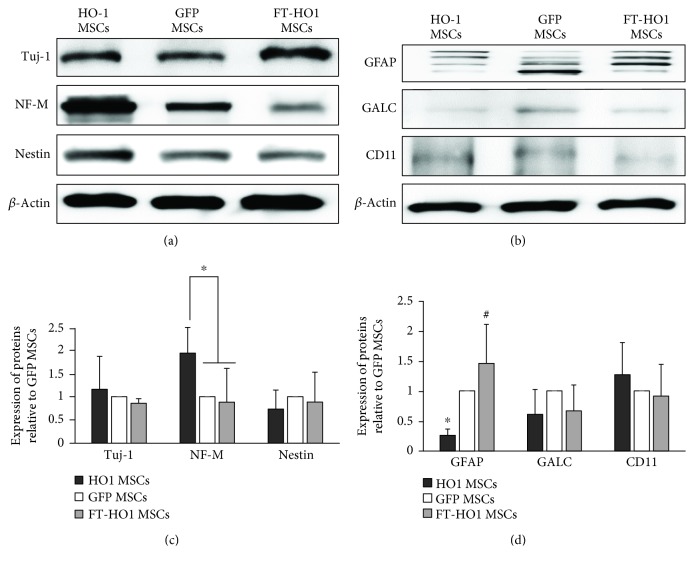
Expression of neural and glial cell markers. (a) Representative densities of neural markers. (b) Representative densities of astroglial cell markers. (c, d) Quantitative analysis of densities obtained for neural and astroglial cell markers relative to GFP MSCs. Each bar represents the average of 4 samples in a group and error bars represent standard error. ∗ and # indicate significance at *p* ≤ 0.05.

The expression of NF-M was significantly higher in the HO-1 MSC group than in the GFP MSC and FT-HO-1 MSC groups (Figure 5, ^∗^*p* ≤ 0.05). Similarly, the expression of GFAP was significantly reduced in the HO-1 MSC group compared with the GFP MSC and FT-HO-1 MSC groups (Figure 5, ^∗^*p* ≤ 0.05). The expression of NF-M was similar between the GFP MSC and FT-HO-1 MSC groups. However, the expression of GFAP was significantly higher in the FT-HO-1 MSC group than in the GFP MSC and HO-1 MSC groups (Figure 5, ^#^*p* ≤ 0.05). No statistical significance was detected when comparing the expression of Tuj-1, nestin, GALC, and CD11 among the groups (Figure 5).

**Figure 6:**
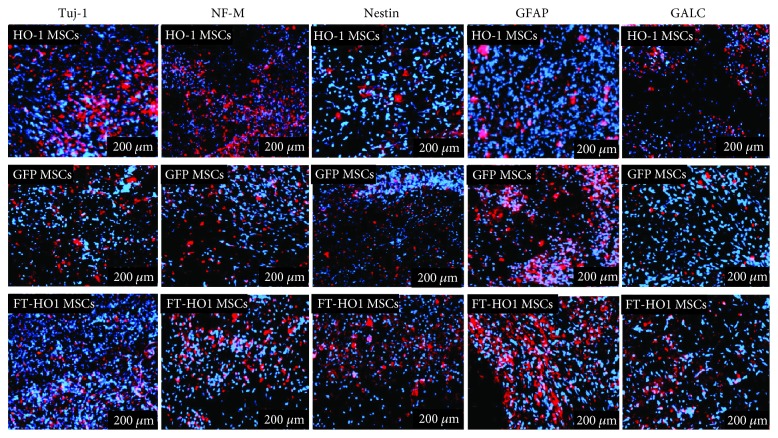
Immunofluorescent staining for the expression of neural (Tuj-1, NF-M, and nestin) and glial (GFAP, GALC) cells. Sections were stained with Tuj-1, NF-M, nestin, GFAP, and GALC in red, and nuclei were stained with DAPI in blue. Each image represents 4 samples per group with a scale bar of 200 *μ*m.

The expression of various neural and glial markers was further confirmed by immunocytochemistry. The expression of these markers followed a similar pattern as what was found with western blot technique. The percent expression of Tuj-1 was similar between all groups, at 16.8% ± 2.14, 10.7% ± 6.14, and 13.23% ± 5.40 in the HO-1 MSC, GFP MSC, and FT-HO-1 MSC groups, respectively (Figure 6). Similarly, the expression of nestin was similar in all groups, at 12.07% ± 0.46, 9.33% ± 2.23, and 10.03% ± 2.33 in the HO-1 MSC, GFP MSC, and FT-HO-1 MSC groups, respectively (Figure 6). The percent expression of NF-M was higher in the HO-1 MSC group at 14.50% ± 2.30 compared to the GFP MSC and FT-HO-1 MSC groups, which were 9.33% ± 2.03 and 10.03% ± 2.33, respectively (Figure 6, *p* ≤ 0.05). Regarding the expression of glial markers, the expression of GFAP was significantly reduced in the HO-1 MSC group (10.87% ± 3.30) compared to the GFP MSC group (14.92% ± 4.14) (Figure 6, *p* ≤ 0.05). However, the expression was significantly higher in the FT-HO-1 MSC group (16.82% ± 2.14) compared to the GFP MSC group (Figure 6, *p* ≤ 0.05). The GALC expression was not significantly affected, at 8.67% ± 2.30, 6.24% ± 3.09, and 9.29% ± 1.34 for the HO-1 MSC, GFP MSC, and FT-HO-1 MSC groups, respectively (Figure 6).

### 3.5. Immune Modulation

**Figure 7:**
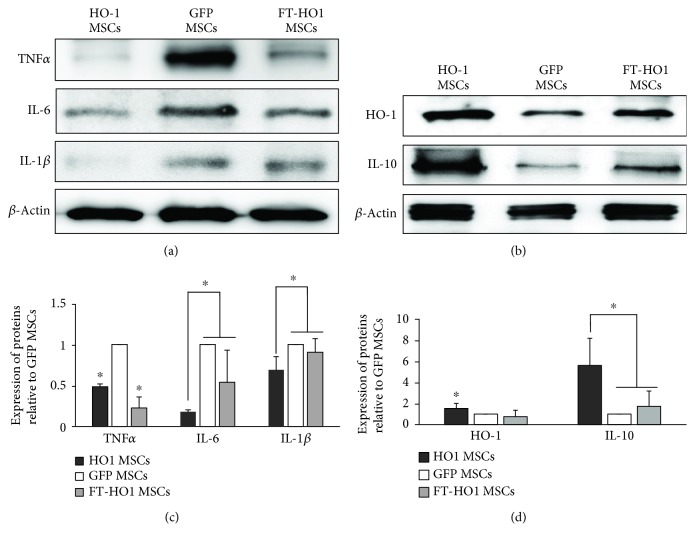
Expression of inflammatory and anti-inflammatory cytokines. (a) Representative densities of markers for inflammatory cytokines. (b) Representative densities of markers for anti-inflammatory cytokines. (c, d) Quantitative analysis of densities obtained for markers related to inflammatory and anti-inflammatory cytokines. Each bar represents the average of 4 samples per group and error bars represent standard error. ^∗^Indicates significance at *p* ≤ 0.05.

The fresh HO-1 MSC group showed a robust immune suppression at the injured segment of the spinal cord after migration. The expression of TNF-*α* was significantly lower in the HO-1 MSC and FT-HO-1 MSC groups (Figure 7, ^∗^*p* ≤ 0.05) than in the GFP MSC group. The expressions of IL-6 and IL-1*β* were significantly lower in the HO-1 MSC group compared to the GFP MSC and FT-HO-1 MSC groups (Figure 7, ^∗^*p* ≤ 0.05); however, the expression was not significantly different between the GFP MSC and FT-HO-1 MSC groups.

### 3.6. Fibrosis and Demyelination

**Figure 8:**
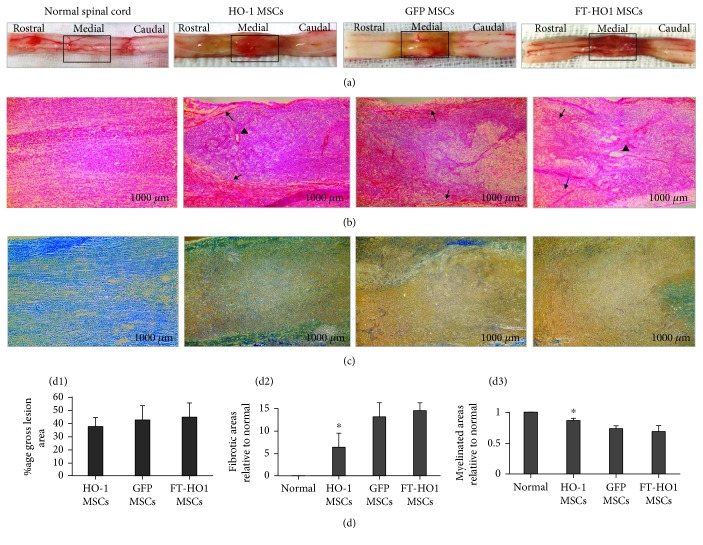
Histopathology of injured spinal cords stained with H&E and luxol fast blue. (a) Injured spinal cord segments with fibrosis, hypotrophy, and hemorrhages. (b) Representative H&E staining of injured spinal cord segments. The fibrotic area is observed as a red & pink color, while the healthy area is observed as purple. (c) Representative staining of myelin with luxol fast blue. Positive staining is depicted by the intensity of the blue color. The images were obtained at low magnification 40x with a scale bar of 1000 *μ*m. (d1) % expression of gross lesion area. (d2) % expression of fibrosis relative to normal. (d3) % expression of the myelinated area relative to normal. Each bar represents the average of 4 samples per group. ^∗^Represents the significance of the HO-1 MSC with the GFP MSC and FT-HO-1 MSC groups at *p* ≤ 0.05. Arrows denote fibrotic areas; arrowheads denote vacuoles.

Upon gross evaluation, the injured area of the spinal cord showed hemorrhages, fibrosis, and atrophy (Figure 8(a)). The extent of the gross lesion area of the injured spinal cord was not significantly different among the groups (Figure 8(d1)). The gross lesion areas quantified were 37.29% ± 6.84, 42.36% ± 10.80, and 44.50% ± 10.70 for HO-1 MSCs, GFP MSCs, and FT-HO-1 MSCs, respectively.

As detected by H&E staining, fibrosis was significantly reduced in the HO-1 MSC group compared with the GFP MSC and FT-HO-1 MSC groups (Figures 8(b) and 8(d2)) (^∗^*p* ≤ 0.05). The degree of fibrosis was 20.20% ± 5.0, 41.80% ± 10.04, and 46.21% ± 5.51 for the HO-1 MSC, GFP MSC, and FT-HO-1 MSC groups, respectively. Fibrosis was mainly confined at the epicenter of the injured area (Figure 8(b) arrow). The gray matter was well preserved in the HO-1 MSC group. Vacuoles were observed in all the groups; however, they were more numerous in the FT-HO-1 MSC group (Figure 8 arrowheads).

As detected by luxol fast blue staining, the injured areas of the spinal cords of the HO-1 MSC group were more deeply stained compared to those of the GFP MSC and FT-HO-1 MSC groups (Figures 8(c) and 8(d3)) (^∗^*p* ≤ 0.05).

## 4. Discussion

It has been previously found that IV-delivered Ad-MSCs improved healing in dogs with acute SCI, compared with nontreated controls [3]. Similarly, dogs with chronic SCI [31] and subacute SCI [32, 33] showed significant improvement in healing after Ad-MSC transplantation compared to nontreated SCI dogs. There are multiple studies in rabbits as well as rats which demonstrate the efficacy of stem cells in the healing of spinal cord injury, through immune modulation, differentiation, and regeneration [15, 34, 35]. As the efficacy of MSCs has been established in different animals, we did not include a negative control (nontreated) group in our study for ethical reasons and considered the GFP MSC group as our control.

In the present study, spinal cord injury was induced through a balloon compression method, chosen based on the simplicity, severity (covering approximately 80% of the space in the spinal canal), and resulting severe compression spinal cord injury [27, 32, 33]. This technique is clinically significant as it closely mimics SCI caused by naturally occurring intervertebral disc herniation (IVDH) in dogs [36].

HO-1 is a stress-induced enzyme which mainly functions to control homeostasis during disease. The antioxidant and anti-inflammatory effects of HO-1 in the treatment of various disease processes have been well documented [6, 37]. Therefore, HO-1-expressing stem cells were produced to deliver increased levels of HO-1 at the injury site. We previously found that HO-1 MSCs have higher antioxidant capacity; however, their proliferation rate was lower than that of Ad-MSCs. They exhibit a broader spindle shape morphology than Ad-MSCs. They showed positive expression of stem cell markers as shown by Ad-MSCs [23]. We found similar physiological characteristics in HO-1 MSCs as those described previously.

It has been previously reported that in dogs with acute SCI, Ad-MSCs migrate to the injured spinal cord segment, lungs, and spleen following IV administration [3]. Similarly, in mice, they migrated into the injured spinal cord, lungs, spleen, and liver after early IV administration [38]. In the present study, we found that HO-1 MSCs also migrated to the injured spinal cord segments and produced increased levels of HO-1 in vivo. The localization and degree of migration of cells were not significantly different among the HO-1 MSC, GFP MSC, and FT-HO-1 MSC groups. The GFP expression was 4-12% 4 weeks after cell transplantation, which is higher than previously reported expressions of 0.002% or 0.029% in mice after 1 week of cell transplantation [38, 39]. In various reports using murine models, no cells were observed 6 weeks after IV transplantation [40] and 4 weeks after transplantation through the subarachnoid space [41]. This is likely because we transplanted a higher number of cells repeatedly for 3 consecutive days. Another reason may be species differences. It can be assumed that repeated transplantation of cells would increase migration of cells at the injured site and would promote antioxidant and anti-inflammatory processes.

In our previous studies, we found that acute SCI dogs injected IV with Ad-MSCs showed more improvement in hind limb locomotion after one week than the control [3]. Similarly, in dogs with subacute SCI model, intraparenchymal transplantation of HO-1 MSCs significantly improved hind limb functions after 7 weeks of transplantation compared to Ad-MSC-injected dogs [16]. In the present study, we found more rapid recovery in 4 weeks than the previously reported 7 weeks of cell transplantation. This may indicate that IV transplantation of cells in the acute stage, i.e., immediately after SCI, could be more effective in controlling the aggression of secondary SCI, and the HO-1 MSCs perform better than MSCs.

Vascular disruption due to SCI causes neuronal ischemia which causes mitochondria to produce an excessive amount of reactive oxygen species (ROS). Increased leukocyte infiltration and intracellular accumulation of calcium further increase the production of ROS. ROS have a significant deleterious role in secondary SCI [42]. They cause lipid peroxidation of the cell membrane, which further produces neurotoxic compounds such as 4-hydroxynenonal (4HNE). Meanwhile, they cause degradation of proteins (lysine, arginine, and histidine) to form protein carbonyl moieties and nitrotyrosine compounds (3NT) [43]. A concomitant infiltration of leukocytes causes increased production of proinflammatory cytokines such as TNF-*α*, IL-6, and IL-1*β*, which induce neuronal apoptosis by activating the caspase pathways [44]. Hence, it is crucial to measure oxidation and inflammation during the early stages of the disease in order to promote neuronal sparing. Ad-MSCs have both antioxidant and anti-inflammatory functions, with improved neural sparing after early IV administration in dogs with acute SCI [3]. In various clinical disorders, transplantation of HO-1 MSCs showed more anti-inflammatory [45, 46] and antioxidant effects [47] than MSCs. We found that the HO-1 MSC group showed more immunosuppression (i.e., reduced expression of TNF-*α*, IL6, and IL-1*β*) and more neuronal sparing. Considering the antioxidant effects of HO-1, an early antioxidant effect in the HO-1 MSC group can also be assumed. Our results corroborate our previous study in dogs with subacute SCI, in which HO-1 MSCs were injected directly into the spinal cord parenchyma [13]. Thus, HO-1 MSCs have a similar function after IV transplantation in acute SCI dogs as that observed following intraparenchymal injection; however, the IV route has additional advantages over an intrathecal injection, as it is simple and rapid and avoids intraspinal hemorrhages.

In rats with SCI, human umbilical cord MSCs reside in the spleen and upregulate the expression of IL-10 in blood plasma. This systemic immunosuppression has neuroprotective and vasoprotective effects [38]. We found that IV administration of HO-1 MSCs in dogs with SCI upregulated the local expression of IL-10 and HO-1 more than GFP MSCs. However, the impact of HO-1 MSCs upon systemic immune modulation requires further detailed investigation.

The spinal cord injury disrupts the blood-brain barrier which permits the infiltration of leukocytes at the injured spinal cord segment. Leukocytes and the resident glial cells build a glial scar which produces a copious amount of inhibitory factors such as chondroitin sulfate proteoglycans (CSPG) and matrix metalloproteinase, which subdue axonal regeneration [48]. Inhibiting the formation of glial scar has been the strategy to ameliorate the hostile environment for neuroregeneration [49]. We found a reduced expression of GFAP in the HO-1 MSC group which may depict a reduced infiltration of astrocytes and a scar formation, and it could be the contributing factor towards improved healing and functional outcomes in the HO-1 MSC group.

In one group, we injected frozen thawed HO-1 MSCs, because the cryopreservation of stem cells allows instant use when required, controlling the progress of the disease during secondary spinal cord injury. Previously, the cryopreserved canine olfactory bulb cells were capable of promoting the axonal regeneration and remyelination in the adult rat central nervous system [50]. We used cryopreserved HO-1 MSCs because HO-1 MSCs have a higher antioxidant capacity; however, the viability of FT-HO-1 MSCs was lower than that of fresh HO-1 MSCs [23]. In the present study, we found that FT-HO-1 MSCs successfully migrated to the injured spinal cord segments, although they were not as physiologically active as HO-1 MSCs despite a higher number being transplanted. This indicates that freeze thawing procedures negatively influenced the biodistribution and homing properties of transplanted cells in vivo [51]. Moreover, FT-HO-1 MSCs did not produce sufficient amounts of anti-inflammatory cytokines and did not efficiently suppress the expression of proinflammatory cytokines as was observed with fresh HO-1 MSCs. This may indicate deleterious effects of freeze thawing procedures upon the expression of certain genes [52]; however, we previously found in vitro that the gene expression of HO-1 was not significantly affected and that FT-HO-1 MSCs produced higher amounts of HO-1 as did by fresh HO-1 MSCs, although their antioxidant capacity was reduced [23]. In the present study, the poor expression of HO-1 by FT-HO-1 MSCs in vivo might be due to a higher fragility of FT-HO-1 MSCs, reducing their ability to fit into the microenvironment where they migrated and further reducing their viability and gene expression [52]. Plenty of data describe how cryopreservation and thawing negatively affect the physiological properties of MSCs. With respect to genetically modified MSCs, a detailed comparative study comparing wild-type and transgenic stem cells in vitro, as well as in vivo, is needed to establish methodologies to produce stem cells resistant to freeze thawing procedures and unfavorable microenvironments of the target organs.

## 5. Conclusion

In the present study, we found that fresh HO-1 MSCs were more efficacious in promoting functional recovery in dogs with acute SCI by reducing astrocyte infiltration and proinflammatory cytokines and hence can be used as a viable alternative to Ad-MSCs and MPSS. The frozen thawed HO-1 MSCs functioned in the same manner as did fresh MSCs and could act as a readily available tool for the emergency treatment of acute SCI.

## Figures and Tables

**Table 1:** Primers for quantitative real-time polymerase chain reaction.

Genes	Primer sequence (5′-3′)	
	Forward	Reverse
GAPDH	CATTGCCCTCAATGACCACT	TCCTTGGAGGCCATGTAGAC
NANOG	CCTGCATCCTTGCCAATGTC	TCCGGGCTGTCCTGAGTAAG
OCT-4	GTCACCACTCTGGGCTCTCC	TCCCCGAAACTCCCTGCCTC
SOX2	GTCCAGCACTACCAGAGCG	CTTACTCTCCTCCCATTTCC
IL-6	TTTTCTGCCAGTGCCTCTTT	GGCTACTGCTTTCCCTACCC
TNF-*α*	ACCACACTCTTCTGCCTGCT	TGGAGCTGACAGACAACCAG
HO-1	GCGTCGACTTCTTCACCTTC	GGTCCTCAGTGTCCTTGCTC
IL-10	CCTGGGAGAGAAGCTCAAGA	TGTTCTCCAGCACGTTTCAG
BDNF	ATGACCATCCTTTTCCTTAC	GATAGAAGGGGAGAATTACC

GAPDH: glyceraldehyde 3-phosphate dehydrogenase; NANOG: nanOG; OCT-4: octamer-binding transcription factor; SOX2: SRY- (sex-determining region Y-) box 2; IL-6: interleukin 6; TNF-*α*: tumor necrosis factor alpha; HO-1: hemeoxygenase-1; IL-10: interleukin 10; BDNF: brain-derived neurotrophic factor.

## Data Availability

The data used to support the findings of this study are included within the article.
